# Disentangling Ancient Interactions: A New Extinct Passerine Provides Insights on Character Displacement among Extinct and Extant Island Finches

**DOI:** 10.1371/journal.pone.0012956

**Published:** 2010-09-23

**Authors:** Juan Carlos Rando, Josep Antoni Alcover, Juan Carlos Illera

**Affiliations:** 1 Departamento de Biología Animal, Universidad de La Laguna, La Laguna, Tenerife, Spain; 2 Institut Mediterrani d'Estudis Avançats, CSIC-UIB, Esporles, Mallorca, Spain; 3 Island Ecology and Evolution Research Group, IPNA-CSIC, La Laguna, Tenerife, Spain; 4 Instituto Cantábrico de Biodiversidad, CSIC-UO-PA, Oviedo, Spain; University of Liverpool, United Kingdom

## Abstract

**Background:**

Evolutionary studies of insular biotas are based mainly on extant taxa, although such biotas represent artificial subsets of original faunas because of human-caused extinctions of indigenous species augmented by introduced exotic taxa. This makes it difficult to obtain a full understanding of the history of ecological interactions between extant sympatric species. Morphological bill variation of *Fringilla coelebs* and *F. teydea* (common and blue chaffinches) has been previously studied in the North Atlantic Macaronesian archipelagos. Character displacement between both species has been argued to explain bill sizes in sympatry. However, this explanation is incomplete, as similar patterns of bill size have been recorded in *F. coelebs* populations from islands with and without *F. teydea*.

**Methodology/Principal Findings:**

The discovery of a new extinct species in Tenerife (Canary Islands), here named *Carduelis aurelioi* n. sp. (slender-billed greenfinch), provides the opportunity to study ancient ecological interactions among Macaronesian finches. To help understand the evolutionary histories of forest granivores in space and time, we have performed a multidisciplinary study combining: (1) morphological analyses and radiocarbon dating (11,460±60 yr BP) of the new taxon and, (2) molecular divergence among the extant finch species and populations in order to infer colonization times (1.99 and 1.09 My for *F. teydea* and *F. coelebs* respectively).

**Conclusion/Significance:**

*C. aurelioi*, *F. coelebs* and *F. teydea* co-habited in Tenerife for at least one million years. The unique anatomical trends of the new species, namely chaffinch-like beak and modified hind and forelimbs, reveal that there was a process of divergence of resource competition traits among the three sympatric finches. The results of our study, combined with the presence of more extinct greenfinches in other Macaronesian islands with significant variation in their beak sizes, suggests that the character displacement has influenced patterns of divergence in bill size and shape on other Macaronesian islands as well.

## Introduction

Resource competition between sympatric species can produce morphological differences between those species, often resulting in ecological divergence. Such interactions frequently result in changes in species' morphology, locomotion patterns and body size of species [1]–[5]. This evolutionary process is known as ecological character displacement [1]. Character displacement provides a unifying framework for understanding the evolutionary mechanisms of species coexistence and how diversity is maintained [6]. However, the past ecological interactions putatively causing character displacement are hard to understand when native elements involved in such interactions have become extinct.

Evolutionary studies on the morphological variation and genetic divergences of insular biotas are mainly based on extant species. However, many extant island biotas represent modified assemblages of the original ones due to losses of native species and additions of exotics by humans [7]–[10]. Among vertebrates, the highest record of insular extinctions during the last millennia due to human actions occurs in birds. The most dramatic examples arise from Hawaii, tropical Pacific Islands and New Zealand, where extinction levels of endemic and native species have reached values of 30–75% [7]–[10].

The bill morphology of avian granivores is a key subject for palaeoecological research. Food choice by avian granivores is determined by seed processing speed, which is related to the different size and shape of both beaks and seeds [5], [11], [12]. The range of preferred seed sizes is related to bill size, especially bill depth, but not to the body mass [11]. Bill morphology in avian granivores has also been an excellent model for the study of adaptive radiation, especially on island populations [4], [5].

*Fringilla teydea* (blue chaffinch) and *F. coelebs* (common chaffinch) provide one of the best examples of diversification within birds in the Macaronesian Islands (North Atlantic Ocean, Figure S1). Morphological variation of *F. coelebs* has been widely studied. The Macaronesian populations have larger body size, shorter wings, and bigger legs and beaks than their mainland relatives [13], [14]. Within Macaronesia, *F. coelebs* are genetically and morphologically differentiated [15], [16]. However, the reasons behind variations in *F. coelebs* bill morphology are still poorly understood. The Azores populations have beak morphology comparable to *F. teydea*, with deeper and wider beaks than their relatives from Madeira and the Canary Islands. Moreover, the average phenotypic variances in bill and skeletal characters of Azorean F. coelebs are approximately 1.5 times higher than in the Madeira and the Canary common chaffinch populations [14]. This morphological divergence has been argued to be the result of ecological character displacement between *F. teydea* and *F. coelebs* [13], [17]. The absence of the former in the Azores could explain why the bill morphology of the *F. coelebs* evolved in the *F. teydea* direction. However, the *F. coelebs* bill morphology in Madeira, an archipelago also without blue chaffinches, is similar to the Canary Island common chaffinches and, consequently, does not fit to this framework. A potential explanation for this apparent contradiction is that a previous, but now extinct, *F. teydea* population was once present in Madeira [17].

The presence of the two chaffinch species in the Canary Islands has been suggested to be a consequence of two different colonization waves, *F. teydea* being the product of the first [13], [14], [17]. The chaffinch species inhabit different Canary forest environments: *F. teydea* is restricted to the Canary Pine (*Pinus canariensis*) forest, located between about 1,300 and 2,000 meters above sea level; and *F. coelebs* occurs mainly on laurel and to a lesser extent on broad-leaved and mixed pine woodlands, situated at lower altitudes (about 500–1,300 meters a.s.l.) and containing the highest richness of plants, supporting a great variety of trees and seeds [18]. *F. teydea* has a diet adapted to the exploitation of pine nuts, with a larger beak (longer, deeper and wider) than *F. coelebs* [13]. The common chaffinch mainly feeds on a variety of invertebrates, as well as laurel forest seeds [19].

The bill morphology and trophic ecology of the other extant forest finches from Macaronesia are well known. *Pyrrhula murina* (azores bullfinch; endemic from São Miguel island and confined to the largest remaining fragment of laurel forest) has shorter but wider beaks than chaffinches and greenfinches [13], [20], [21], and has a diet based on fleshy-fruits, seeds and, an unusual component in the diet for a bird, fern sporangia [20]. The beak of *Serinus canaria* (wild canary), an endemic to the Azores, Madeira and the Canary Islands that inhabits semi-open habitats with small trees, is smaller than the beak of *F. coelebs*, and they feed on small seeds and occasionally small insects [19], [22]. Finally, *Carduelis chloris* (common greenfinch), which arrived in Macaronesia within the last 40 years, and inhabits mainly farmlands and urban sites, feeds on seeds and occasionally on invertebrates [19], [22].

The extinct finch record obtained from Upper Pleistocene-Holocene paleontological sites in Macaronesia include: 1) an undescribed extinct species of *Carduelis* sp. and *Coccothraustes coccothraustes* (hawfinch), both reported in the fossil record of Madeira [23]; 2) *Carduelis triasi* (Trias greenfinch), an extinct greenfinch from La Palma (Canary Islands) [24] and 3) fragmented bones related to *C. chloris* (common greenfinch) have been described in the fossil record of La Gomera [25]. Unfortunately, no data exist about the diet of these extinct species.

In this paper we use the discovery of a new extinct greenfinch species, named here *C. aurelioi* (slender-billed greenfinch), which inhabited forest habitats in Tenerife, to infer some ancient ecological interactions among Macaronesian forest finches. In particular, we perform a multidisciplinary approach studying: 1) the morphology of the new extinct greenfinch, comparing it with the extant finches; 2) the extinction date of the new species using an AMS ^14^C dating from the collagen of its bones; and 3) the colonization times of extant chaffinch species inferred from sequences of the mitochondrial cytochrome b gene.

We show how an extinct species, in combination with molecular and geological information, can help us to understand patterns in morphology of the extant ones. These results also provide insights on past relationships among other sympatric finch species inhabiting Macaronesia, giving a new perspective on the evolutionary and biogeographic history of the avifauna in the region.

## Results

### Systematic Palaeontology

Order Passeriformes

Family Fringillidae

Genus *Carduelis* Bisson, 1760

Materials could be attributed to the genus either *Carduelis* or *Serinus* based on the following combination of cranial characters: strong and vigorous maxilla and mandible, *processus zygomaticus* and *postorbitalis* very well developed, *processus palatinus premaxillaris* not fused with the *palatinum* and forming a lateral flange, only one *fonticulum orbitale*, a single and long *foramen orbitonasale*, *foramen venae occipitalis externae* at the posterior edge of *foramen magnum*, and a long *processus orbitalis quadrati*. However, it differs from *Serinus* in the larger overall size, in the wider morphology of the ventral lobe of *os ectethmoidale* and the wider transpalatine process.

**Figure 1:**
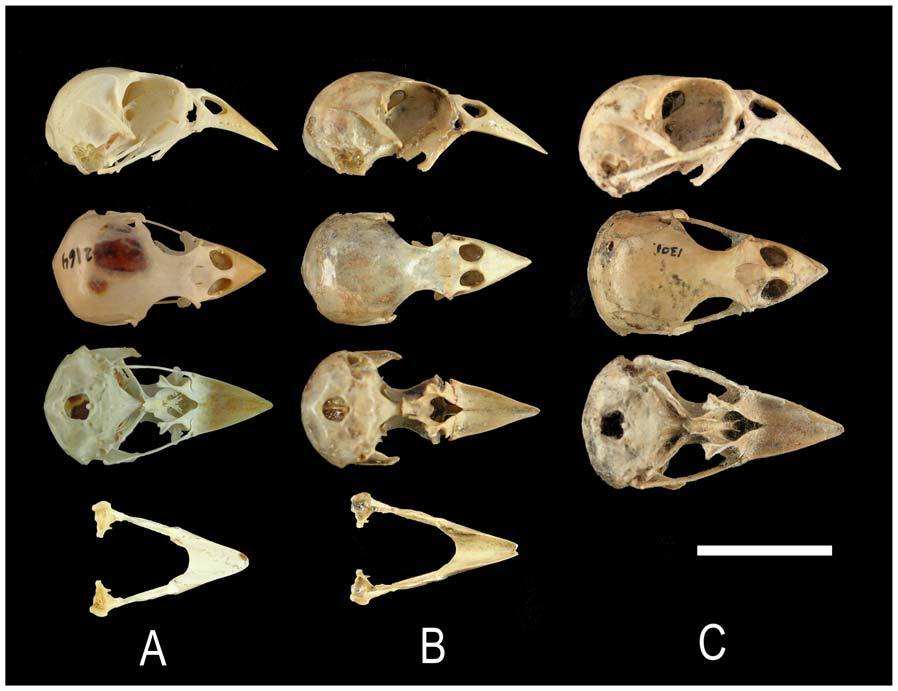
Comparison of cranium and mandible of *Carduelis aurelioi n. sp.* Cranium and mandible of *Carduelis aurelioi* (B) , DZUL 3047 Holotype, *C. chloris* (A), IMEDEA 2164, and *C. triasi* (C), DZUL 1301. From up to down: cranium, right lateral, dorsal and ventral views, and mandibles dorsal views. The mandible of *C. triasi* has not yet been found. Scale = 2 cm.

**Figure 2:**
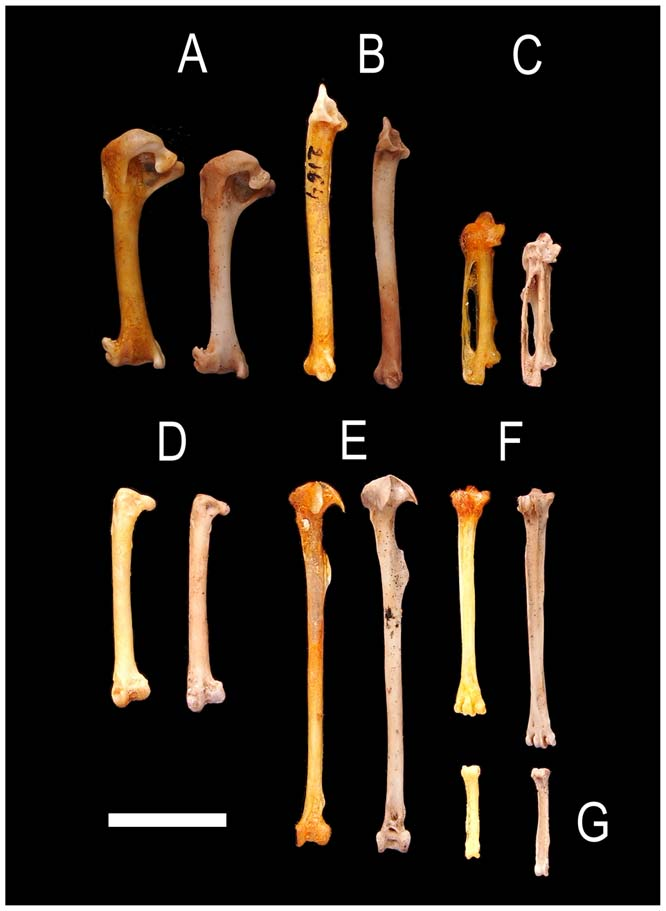
Comparison of wing and leg bones of *Carduelis aurelioi n. sp.* Wing and leg bones of *Carduelis aurelioi* (right series, several specimens combined) and *C. chloris*, IMEDEA 2164 (left series). (A) humeri, caudal view; (B) ulna, ventral view; (C) carpometacarpi, ventral view; (D) femora, caudal view; (E) tibiotarsi, cranial view; (F) tarsometatarsi, plantar view; (G) pes phalanx 1 of digit I, plantar view. Scale = 1 cm.

**Figure 3:**
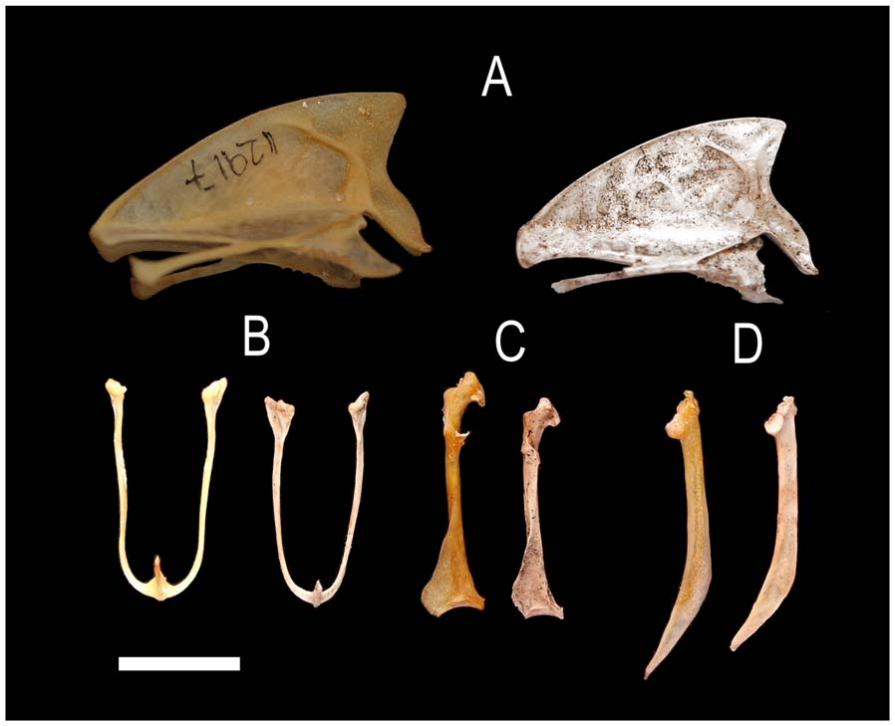
Comparison of skeletal elements of *Carduelis aurelioi n. sp.* Skeletal elements of *Carduelis aurelioi* (right series, several specimens combined) and *C. chloris*, IMEDEA 12917 (left series). (A) Sternum, left lateral view; (B) clavicula, caudal view; (C) coracoideum, dorsal view; (D) scapula, left lateral view. Scale = 1 cm.

***Carduelis aurelioi***, new species (Figures 1, 2 and 3)

urn:lsid:zoobank.org:act:15B12DEA-B918-4C3D-8524-1702FCD286FA

Associated bones of 12 specimens are known. One of them, the most fragmented skeleton, was used to perform radiocarbon dating (a destructive analysis).

#### Holotype

Specimen 1 (DZUL 3047): associated skeleton including complete cranium lacking jugal bars, complete mandible (Figure 1B), right quadratum and complete right tarsometatarsus.

#### Type locality

Cueva del Viento.

#### Distribution

Tenerife Island, Canary Islands.

#### Horizon

Upper Pleistocene – Holocene: 11,460±60 yr BP (13,427–13,207 cal yr BP; lab code: KIA-30992) is the ^14^C age obtained for the sole dated specimen.

#### Status

Extinct.

#### Etymology

The specific name is in honour of Professor Aurelio Martín (Department of Zoology from La Laguna University) because of his numerous and impressive contributions to the knowledge and conservation of vertebrates in the Canary Islands.

#### Paratypes

Specimen 2 (DZUL 3048): associated elements including complete cranium lacking palatal area and jugal bars, complete mandible, both palatines, both pterygoids, fragment of right humerus, fragment of right radius, right tibiotarsus (lacking proximal part), and both tarsometatarsi.

Specimen 3 (IMEDEA 90647): associated elements including complete cranium lacking jugal bars, 4 fragment of mandible, both near complete coracoids, 2 fragments of left scapula (including facies articularis humeralis), 2 fragments of right scapula, 3 fragments of stermun (including manubrium sterni), fragment of synsacrum (axial part), both humeri, near complete left ulna, two fragments of radius, distal fragment of right tibiotarsus, and right tarsometarsus (lacking distal part).

Specimen 4 (IMEDEA 90648): near complete cranium lacking palatal area and jugal bars.

Specimen 5 (IMEDEA 90649): associated elements including complete cranium lacking jugal bars (with right quadratum fused to the braincase), near complete mandible (2 fragments), left scapula, left coracoid, proximal part of right coracoid, near complete sternum (including manubrium and carina sterni), sinsacrum (axial part), near complete left isquium, ilium an pubis, both humeri, left ulna, right carpometacarpus, both femora, right tibiotarsus, left tibiotarsus (lacking distal part), both tarsometatarsi and 1 vertebra.

Specimen 6 (DZUL 3049): associated elements including complete cranium lacking jugal bars, both fragments of jugal bars, complete mandible, both pterygoids, both coradoids and scapulae, 4 fragments of sternum (including manubrium sterni and right trabecula lateralis), fragment of clavicula (including apophysis furculae), synsacrum (axial part), complete left isquium, ilium and pubis, fragment of right ilium, both humeri, ulnae, radii, and carpometacarpi, left tibiotarsus, both tarsometartarsi, 2 pedal phalanx and 3 vertebrae.

Specimen 7 (DZUL 3050): associated elements including a fragment of braincase lacking palatal area (complete left temporal area, septum interorbilate and area around foramen magnum), near complete maxilla, left quadrate, near complete mandible (2 fragments), right scapula, both coracoids, right humerus, left humerus (lacking distal part), left ulna, both carpometacarpi, fragment of sternum (proximal part including manubium sterni), synsacrum (axial part), fragment of right ilium, isquium and pubis (including foramen acetabuli and foramen ilioischiadicum), left femur, right femur (lacking distal part), both tibiotarsi and tarsometatarsi.

Specimen 8 (IMEDEA 90650): associated elements including near complete cranium lacking, maxilla, jugal bars and palatal area, complete mandible, left quadrate, near complete left scapula, near complete sternum, near complete synsacrum (2 fragments) including ilium, isquium and pubis, both humeri, ulnae, and radii, left carpometacarpi, both tibiotarsi (fragmented), right tarsometatarsi, and 2 vertebrae.

Specimen 9 (DZUL 3051): associated elements including a fragment of cranium lacking, maxilla, jugal bars, palatal and left parietal area, near complete maxilla, near complete mandible (2 fragments), right coracoid, proximal fragments of both scapulae, synsacrum (axial part), fragment of stermum (including manubrium sterni and apex carinae), both humeri, ulnae, and carpometacarpi, left radium, right femur, left tibiotarsus and both tarsometatarsi.

Specimen 10 (DZUL 3052): associated elements including both quadrates and palatinum, both coracoids, fragments of left scapula, complete clavicula and sternum, 3 fragment of synsacrum including ilium, isquium and pubis, both humeri, ulnae, radii and carpometacarpi, left femur, 2 fragments of right femur, both tibiotarsi, and 5 vertebrae.

Specimen 11 (DZUL 3053): associated elements including fragment of cranium lacking dorsal braincase, palatal area and jugal bars, fragment of maxilla, left quadrate, near completa mandible (2 fragments), fragment of sternum (including manubrium sterni), right humerus, ulna, and carpometacarpus, left tibiotarsus (2 fragments) and both tarsometarsi.

#### Institutions housing material

Holotype (specimen 1) and Paratypes (specimens 2, 6, 7, 9, 10 and 11) Vertebrate Collection of Departamento de Biología Animal (Zoología), at the Universidad de La Laguna, La Laguna, Tenerife, Canary Islands, Spain (DZUL 3048-3053); Paratypes (specimens 3, 4, 5 and 8), Institut Mediterrani d'Estudis Avançats (CSIC-UIB), Mallorca, Balearic Islands, Spain (IMEDEA 90647–90650).

#### Common name proposed

slender-billed greenfinch.

#### Diagnosis

The new species differs from extant species of *Carduelis* in cranial features as follows: cranium longer with a stronger *processus zygomaticus*; bigger *processus postorbitalis* and laterosphenoidale crests, stronger robust palatine especially transpalatine process; longer beak, more conical, with the distal part smoothly compressed laterally. It differs from extinct *Carduelis triasi*, as follows: cranium smaller; beak shorter, less robust; *processus zygomaticus* and palatine less robust [24] (Figure 1).

Postcranial osteological features are rather homogeneous in the genus *Carduelis*. However, some characteristic features can be identified in comparison to *C. chloris*. In the new taxon the wing bones are shorter, the ulna has a smaller *cotyla dorsalis* and *condylus dorsalis*; the coracoid shows a smaller *facies articularis humeralis*, and a shorter *facies articularis sternali*s; the scapula has a weak *facies articularis humeralis*, the sternum has a smaller *carina* and *rostrum sterni*. The tibiotarsus has a *crista cnemialis lateralis* and *crista cnemialis cranialis* larger in the new taxon. In addition, the femur is shorter and the tibiotarsus is longer in the new species (Table 1, Figures 2 and 3).

**Table 1:** Bone measurements of greenfinches species.

Morphological traits	*C. triasi*	*C. aurelioi* n.sp.	*C. chloris*	%	*p*
1: Maxilla length	19.10 (1)	16.95±0.69 (4)	15.13±0.55 (26)	+12	<0.005^m^
2: Maxilla width	9.67 (1)	7.96±0.3 (7)	8.42±0.45 (26)	−5.5	ns^m^
3: Maxilla height	6.71 (1)	5.17±0.15 (6)	5.20±0.23 (26)	−0.6	ns^m^
4: Interorbital width	6.11 (1)	4.91±0.4 (10)	5.76±0.37 (27)	−14.7	<0.05^m^
5: Cranium width	17.47 (1)	15.62±0.36 (6)	15.34±0.48 (7)	+ 1.8	ns^u^
6: Cranium length	34.89 (1)	32.3±1.04 (3)	29.76±0.61 (11)	+ 7.9	<0.05^u^
7: Cranium height	14.31 (1)	12.94±0.51 (7)	12.65±0.28 (11)	+ 2.2	ns^u^
8: Mandible length		24.27±1.53 (6)	22.94±0.92 (24)	+5.8	= 0.005^m^
9: Symphysis length		8.83±0.58 (6)	8.14±0.46 (24)	+8.5	<0.05^m^
10: Mandible width		15.92±0.78 (4)	15.72±0.62 (10)	+1.2	ns^u^
11: Articular end width		4.96±0.36 (7)	5.38±0.23 (20)	−7.8	<0.05^m^
12: Mandible height		5.94±0.5 (8)	6.12±0.42 (22)	−2.9	ns^m^
13: Scapula length		19.62±0.53 (2)	22.68±0.57 (22)	−13.5	<0.05^u^
14: Coracoid length		16.42±0.66 (6)	18.23±0.5 (25)	−9.9	<0.005^m^
15: Humerus length		17.49±0.64 (8)	18.55±0.37 (25)	−5.7	<0.005^m^
16: Ulna length		20.81±0.7 (6)	23.21±0.64 (26)	−10.3	<0.005^m^
17: Carpometacarpus length	11.69 (1)	11.46±0.43 (7)	13.37±0.41 (26)	−14.3	<0.005^m^
Total wing bones length		49.78±1.81 (6)	55.14±1.31 (25)	−9.7	
18: Femur length		16.32±0.77 (4)	17.20±0.33 (26)	−5.1	<0.05^u^
19: Tibiotarsus length		27.39±1.14 (5)	27.22±0.72 (24)	+0.6	ns^m^
20: Tarsometatarsus length		19.24±0.8 (8)	17.61±0.54 (26)	+9.3	<0.005^m^
Total leg bones length		61.54±2.71 (3)	62.02±1.49 (23)	−0.8	
21: Basal Phalax Dig I length		8.25 (1)	7.03±0.22 (21)	+17.3	
22: Sternum length		20.41±0.68 (3)	23.82±0.59 (22)	−14.3	<0.05^u^
23: Carina sterni height		10.19±0.51 (4)	11.84±0.35 (22)	−13.9	<0.005^u^
24: Area of carina and rostrum sterni		92.88±4.19 (3)	133.89±8.39 (20)	−30.6	<0.05^u^

Mean length ± standard error (mm) and mean area ± standard error (mm^2^) for *Carduelis triasi* (Trias greenfinch), *C. aurelioi* n. sp. (slender-billed greenfinch) and *C. chloris* (common greenfinch). Sample size is shown in brackets. (%): percentage of variation between measurements of *C. aurelioi* and *C. chloris*, and statistical significance from MANOVA (^m^) and from Mann-Whitney U tests (^u^), ns (non significant). Measurements are as in Figure S6.

#### Remarks

The cranial osteology strongly suggests that *C. aurelioi* is closely related to *Carduelis* finches with robust and pyramidally-shaped bills such as *C. chloris* and *C. triasi*. The similarity to these taxa is mainly due to the shape of the braincase, the strong *processus zygomaticus* and the robust morphology of the *postorbitalis*, *ectethmoidale* and palatinum bones, and a similarly shaped narina opening. The *processus palatinus premaxillaris* not fused with the *palatinum* forms an identical lateral flange in these three species. These shared morphological characteristics indicate a very close phylogenetic proximity among these three taxa within the genus *Carduelis*. Differences with *C. carduelis* (goldfinch) and *C. spinus* (siskin) finches with thin bill, are in overall size, the morphology of the palatinum bone, and the strength of the *processus zygomaticus*. In spite of the similarities with *C. chloris* and *C. triasi*, differences exist in cranium length, and in the slender bill and mandible of the new taxon when it is compared to *C. chloris* (the mandible of *C. triasi* has never been found). Differences also exist in the size and degree of developing of *processus zygomaticus* and *postorbitalis*, and palatinum bones, symphysis length, the narrow interorbital constriction and postcranial lengths and proportions (Table 1; Figures 1, 2 and 3).

### *Carduelis aurelioi* morphology

Although *C. aurelioi* is a similar size to *C. chloris*, it displays very different anatomical traits. The MANOVA (performed with morphological traits 1–4, 8, 9, 11, 12, 14–17, 19 and 20; Table 1) identified high morphological differences between these species (Wilks' lambda = 0.011, d.f. = 14,9; *p*<0.005). The new bird has a longer beak than *C. chloris* (F_1,24_ = 29.709; *p*<0.005, trait 1). The mandible and the symphysis are longer in the new species (F_1,24_ = 9.509; *p* = 0.005 and F_1,24_ = 7.016; p<0.05, traits 8 and 9 respectively). Overall, the head is 7.9% longer in *C. aurelioi* than *C. chloris* (U = −2.572; *p*<0.05, trait 6). The new species has shorter scapula (U = −2.028; p<0.05, trait 13) and coracoid (F_1,24_ = 38.359; p<0.05, traits 14), and a smaller sternum (length and height; U = −2.761, U = −3.129; p<0.05 respectively for trail 22 and 23) with a great reduction in the area of *carina* and *rostrum sterni* (U = −2.739; p<0.05, trait 24). It also has shorter total length of the wing bones (humerus+ulna+carpometacarpus). Reductions in length of the proximal, central and distal bones are 5.7%, 10.3% and 14.3% respectively (F_1,24_ = 17.467, F_1,24_ = 43.615 and F_1,24_ = 52.514; with *p*<0.005 for the three bones, traits 15, 16 and 17, respectively). Both species shows similar total length of hind limb bones (i.e. femur+tibiotarsus+tarsometatarsus). However, the new species has a shorter femur (U = −2.38; p<0.05) and a longer tarsometatarsus (F_1,24_ = 28.953, *p*<0.005) than *C. chloris*. The only Pes Phalanx 1 of digit I found is remarkably longer in the new taxon (Table 1, Figures 2 and 3).

The MANOVA performed with beak and cranial variables (traits 1–5, 7–12) of granivores forest birds from Tenerife Island (*C. aurelioi*, *F. teydea teydea* and *F. coelebs canariensis*) show strong morphological differences among these birds (Wilks' lambda = 0.042, d.f. = 6,32; *p*<0.005). The PCA analysis performed with the same traits captured two principal components explaining 88% of total variance. Of this total 52.2% is explained by PCA1 which measures length beak and cranium shape, meanwhile PCA2 explained 35.8% of variance which measures width and height beak traits (Figure S2).

The MANOVA performed in order to compare beak variables (traits 1–3) between *F. teydea teydea* (Tenerife) and *F. teydea polatzeki* (Gran Canaria) provided significant differences (Wilks' lambda = 0.153, d.f. = 3,4; *p*<0.005). All beak traits accounted for these differences (F_1,8_ = 23.194, F_1,8_ = 69.350 and F_1,8_ = 14.470; with *p*<0.005 for the 1–3 traits, respectively). Finally, the MANOVA performed with beak traits between *C. aurelioi* and *F. teydea polatzeki* showed significant differences (Wilks' lambda = 0.098, d.f. = 3,4; *p*<0.05). The maxilla length (F_1,8_ = 11.597, p = 0.014) was the only morphological trait accounting for this difference.

We estimated the weight of *C. aurelioi* to be 22.21±2.73 g (n = 5). We tested the validity of the method for nine species of fringillids, comparing the published weights [22], [26] with those derived from tibiotarsus length regression. No significant differences were found between the estimated and measured weights (t = 1.17; *p* = 0.28).

### Flight capacity of *Carduelis aurelioi*

We evaluated flight capability with a comparison of the ratios of combined humerus, ulna and carpometacarpus lengths to femur length [27]–[29]. The values calculated for flighted species of *Carduelis* are 3.3∶1 for *C. cannabina* (linnet) and 3.2∶1 for *C. chloris*. For *C. aurelioi* obtained a ratio of 3.0∶1. The values obtained for some flightless passerines are lower: *Traversia lyalli* (Stephens Island wren; 2.0∶1), *Dendroscansor decurvirostris* (long-billed wren; 2.3∶1), and *Emberiza alcoveri* (long-legged bunting; 2.1∶1) [27]–[29].

We obtained the equations: Y = 1.286 X+15.654 (*p*<0.001, r^2^ = 0.97, n = 11) to estimate wing length; and Y = 119.757 X−2696.603 (*p*<0.004, r^2^ = 0.79, n = 7) to estimate wing area. According to this, wing length of *C. aurelioi* was 79.67±2.33 mm , and wing area 32.66±2.18 cm^2^ (n = 6). Using these estimations, the wing is 8% shorter (86.69±1.58 mm; n = 22) and 16.4% smaller in area than in *C. chloris* (39.19±1.47 cm^2^; n = 22).

We calculated the ratio of body weight to wing area and we obtained a wing loading of 0.34±0.02 g cm^−2^ for *C. aurelioi* (n = 5). This value is about 18% bigger than in *C. chloris* (0.28±0.02 g cm^−2^, n = 20), and around 30% higher than in *Geospiza fortis* (medium ground finch) [30].

**Figure 4:**
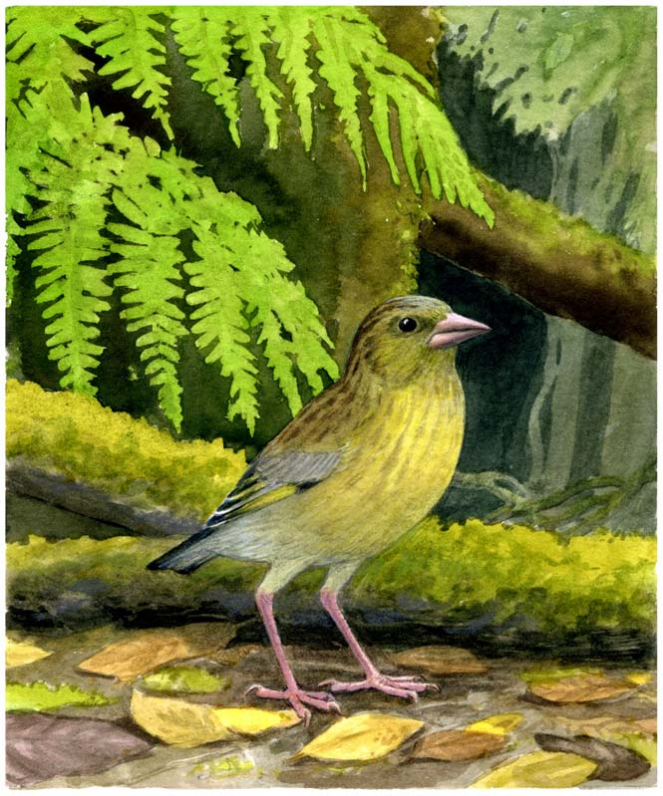
Reconstruction of *Carduelis aurelioi n. sp.* (slender-billed greenfinch) at laurel forest, art by A. Bonner.

The reduced *carina* and *rostrum sterni*, the ratio of combined humerus, ulna and carpometacarpus length to femur length, together with the estimated wing length, wing area and wing loading indicate that *C. aurelioi* was a weak flying passerine. Overall, these results strongly suggest that it was a ground feeder (Figure 4).

### Phylogenetic relationships and colonization times of Chaffinches

We obtained a final alignment of 1002 base pairs from the cyt-b gene (see Table S1 for Genbank accession numbers). The best model selected by Modeltest was the general time reversible model (GTR+G). Uncorrected pairwise distances within populations of *F. coelebs* ranged from 0.1% to 2.0%, and 1.8% between the two *F. teydea* populations (Table S2). Monophyly of *F. teydea* and *F. coelebs* populations receives moderate support with Bayesian Inference (Figure S3, node A). Within this clade all *F. coelebs* individuals are grouped together with high nodal support. The two blue chaffinch subspecies (*F. t. teydea* and *F. t. polatzeki*) are also grouped together with high nodal support (Figure S3). Divergence times obtained from BEAST suggest that *F. teydea* first colonized Macaronesia around 1.99 My ago (Figure S3, node A). The lineage that would give rise to the Macaronesian *F. coelebs* populations is estimated to have diverged from mainland populations approximately 1.28 My ago (node B). The Canary and Madeira *F. coelebs* populations appear to have split around 1.09 My ago (node C). Finally, the diversification of the Canarian *F. coelebs* is estimated to have commenced approximately 0.85 My ago (node D).

## Discussion

### Habitat use of *Carduelis aurelioi*

The proportions of leg bones of *C. aurelioi* (26, 43 and 31% of the total length of leg bones for femur, tibiotarsus and tarsometarsus, respectively) are different from those of C. chloris (28, 44, and 28%), but they are identical to leg length proportions in *F. teydea* and to *E. alcoveri*, an extinct and flightless passerine from Tenerife [29]. These identical proportions probably reflect similar foraging behaviour. *F. teydea* feeds on pine seeds, either picking up them on the ground or extracting them from fallen and opened cones [31]. *C. aurelioi* was probably ground-dwelling as well, although not in the pine forest. It is likely that the dense herb layer typical of the laurel forest close to the fossil site offered protection to weak-flying species from avian predators such as the Eurasian sparrowhawk (*Accipiter nisus*), which has been also recorded in the same fossil site [32], so probably the slender-billed greenfinch mainly inhabited this ecosystem.

### Evolutionary history of Chaffinches and Greenfinches in Macaronesia

The colonization of *F. teydea* an *F. coelebs* in Macaronesia occurred during the Pleistocene epoch; probably with a difference around 700,000 years, which are likely to be associated with Quaternary interglacial periods. The arrival of the ancestor of *F. teydea* to the Canary Islands (≈2 My ago; node A, Figure S3) occurred before El Hierro appeared (1.1±0.02 My) [33], and slightly before La Palma had emerged (1.7±0.2 My) [34], precluding its colonization. The colonization of *F. coelebs* in Macaronesia is estimated to have occurred around 1.28 My ago (node B, Figure S3). The observed pattern is consistent with a colonization event of Macaronesia from north (Azores) to south (Canary Islands), taking advantage of the dominant trade winds blowing in that direction [16] (Figure S3). The split of the Canary-Madeira clade is estimated to have occurred during the last million years (1.09 My ago; node C, Figure S3), a period where La Palma had emerged but El Hierro only was starting to appear. The close phylogenetic affinities between La Palma and El Hierro *F. coelebs* populations suggest that La Palma was the colonization source of El Hierro (node F; Figure S3).

Considering morphological differences between *C. aurelioi* and its closest living relative, *C. chloris*, it is plausible to assume that the initial colonization of the ancestor of this species took place well before the arrival of *F. coelebs* to Macaronesia. Common chaffinches are more similar -morphologically and biometrically - to their mainland relatives than slender-billed greenfinches are. In fact, all Macaronesian common chaffinch populations are considered the same species as European and African mainland populations, in contrast to *F. teydea* and *C. aurelioi* which are morphologically different enough to be recognized as endemic species.

Radiocarbon dating confirms that the slender-billed greenfinch extinction occurred after 13,427 cal yr BP. The combination of this result with the molecular data indicates that the three finches co-occurred in Tenerife for around 1 My (mean value of the sampled trace across the chain). The interpretation of a long time co-occurring is also consistent with the estimated lowest age of colonization of the *F. coelebs* (0.37 My for the lower bound of the 95% highest posterior density interval; node C, Figure S3).

### Character displacement among extinct and extant finches

*Carduelis aurelioi* morphology indicates an evolutionary trend in the direction of a chaffinch-like bill; that is, conically shaped instead of the shorter and wider pyramidal beak typical of other greenfinches. The longer bill in *C. aurelioi* suggests more versatile feeding habits than greenfinch species with robust and pyramidal bills, perhaps including a higher invertebrate component, similar to *F. coelebs* [22], [35]. The bill of *C. aurelioi* is morphologically halfway between that of the *F. coelebs* and *F. teydea* from Tenerife Island (Figure S4). The bill width to length and height to length ratios are close for these three species (0.47, 0.30; 0.42, 0.27; and 0.44, 0.28; for *C. aurelioi*, *F. coelebs*, and *F. teydea*, respectively), but significantly different from the common greenfinch (0.56, 0.34).

Grant did not find any overlap among bill measurements of the sympatric *F. coelebs* and *F. teydea* [13]. However, *C. aurelioi* shows an overlap in bill height with *F. teydea* (Figure S4), a morphological trait directly related to the maximum compression force that the mandibles can apply [36], and the size and hardness of seed that might be used [5], [11]. Differences in beak morphology between *F. coelebs* and *C. aurelioi* suggest that both species mainly fed on different type of seeds within the laurel forest. Because molecular and radiocarbon dating results confirm that the three finches coexisted in Tenerife forests until the recent extinction of *C. aurelioi*, ecological character displacement emerges as the most plausible hypothesis to explain the observed differences in beak size, as this process reduces resource competition between sympatric species of different groups [2], [3]. In this ancient scenario with three finch species coexisting, *C. aurelioi* played probably a key role due to its intermediate beak size. In this system, with species displaying similar bill shapes but different sizes, the central species competes with the other two. However, the interactions with *F. teydea* were probably the strongest due to the more similar shape and size of the beaks between these two species.

The bill morphology of extant chaffinches and recently extinct greenfinches in Macaronesia suggests that the character displacement may not have been an unusual process. In fact, on four (Madeira, Tenerife, La Gomera and La Palma) out of six Macaronesian islands where populations of *F. coelebs* share similar beak size (Madeira, La Palma, El Hierro, La Gomera, Tenerife and Gran Canaria), extinct greenfinch species have been found in the fossil record (Figure S1). The two exceptions are El Hierro and Gran Canaria, although caution is required here, as the palaeornithological record is very scarce on both islands. In the Azores *F. coelebs* is the sole finch species inhabiting the archipelago. There are no fossil records of extinct either *Carduelis* sp. or *F. teydea* species from these islands.

The exclusive occurrence of *F. coelebs* in the Azores would suggest the absence of character displacement in these populations. If so, in the absence of competition, we should find that of all *F. coelebs* populations in Macaronesia, those from the Azores possess the most robust beaks. Grant provided evidence supporting this hypothesis: the Azores chaffinches have the deepest and widest beaks of all Macaronesian *F. coelebs*, with a halfway morphology between *F. teydea* and *F. coelebs* from the Canary Islands (Figure S5) [13]. Bigger bills allow birds to use larger seeds without affecting the profitability of small ones [11]. In addition, and taking into account that variation of beak size in granivore birds is greater in the absence of competition [2], the highest variances detected in bill size in the Azores populations among all Macaronesian populations [14], support the absence of character displacement in Azores (Figure S5).

**Figure 5:**
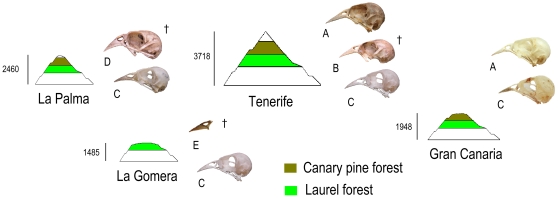
Skull and distribution of forest finches from Canary Islands. Forest finch skulls from Gran Canaria, Tenerife, La Palma and La Gomera (Canary Islands). Extinct (†) and extant species, altitude (m) and forest type present on each island is also showed. (A) *Fringilla teydea*; (B) *Carduelis aurelioi*; (C) *F. coelebs*; (D) *C. triasi*; and (E) *Carduelis* sp.

There are also significant morphological differences among the extinct greenfinches in Macaronesia (Figure 5). The extinct *C. triasi* from La Palma had an, even more robust bill than *F. teydea* (Table 1, Figures 1 and 5). On the other hand, the bill of *C. aurelioi* is more slender. In La Gomera, the only two fragments (skull and premaxillary) found were initially assigned to *C. chloris* [25]. However, a recent review of this material has cast doubts on its taxonomic position, even suggesting that it could be assigned to *C. aurelioi* or to a new *Carduelis* species. As such, this material has been reassigned to *Carduelis* sp. Finally, no data have been published about bill morphology of the extinct greenfinch from Madeira. Thus, the variations observed among extinct greenfinches could be a direct consequence of: (a) the number of granivore bird species coexisting in sympatry; (b) the availability of seed types, which is mainly related with the type of forest.

With regards to the number of finch species inhabiting in sympatry, the most complex situation is observed in Tenerife, where three species coexisted in two types of forests (Canary pine and laurel forests). The bill of *C. aurelioi* shows a halfway morphology between the smaller bill of *F. coelebs canariensis* and the larger *F. teydea teydea* bill all from Tenerife (Figures 5 and S4). The MANOVA performed with beak and cranial variables, and the PCA with the same traits (Figure S2) indicate strong morphological differences among these species.

However, only two species of finch co-occurred in the two types of available forests on La Palma. Here, *C. triasi* beak has evolved towards the largest size, probably due to the absence of *F. teydea*, a direct competitor for hard seeds (Table 1; Figures 5 and S1). *Carduelis triasi* probably mainly inhabited and fed on the laurel forest floor [24]. The bill morphology of this bird allowed the use of a wider range of seeds including pine nuts. A comparable situation was observed and documented by Grant & Grant on Daphne Major (Galápagos archipelago) where *Geospiza fortis* (medium ground finch) possessed bigger beaks in the absence of a direct competitor such as *G. magnirostris* (large ground finch) [4]. Strong selection occurred towards smaller beak sizes in G. fortis after the arrival of *G. magnirostris*, especially after a dry season when food supply was a limiting factor. On the contrary, in La Gomera, where two species of finches cohabited and only laurel forest exists, the bill morphology of the extinct greenfinch is medium size (similar to *C. aurelioi*; Figure 5). The absence of pine woodlands (and hard nuts) in La Gomera could prevent strong directional selection towards large-sized bills.

The above considerations provide the opportunity to make the prediction that on El Hierro an extinct species of greenfinch, with a beak comparable to that of *C. triasi* (i.e. large-sized), is expected to be found because: 1) both forest types are present, and 2) *F. coelebs* is the only chaffinch species inhabiting the island, and has a bill morphology similar to the other Canary and Madeira chaffinch populations. In contrast, on Gran Canaria, where *F. teydea* and *F. coelebs* co-inhabit, the situation seems to be more complex. In Gran Canaria, *F. coelebs* is not morphologically different from the rest of Canary populations [13]. However, *F. teydea polatzeki* (Gran Canaria) shows a smaller beak than the *F. teydea teydea* (Tenerife). Opposite, *F. teydea polatzeki* and *C. aurelioi* do not show significant differences in bill width and bill height (Figure 5 and S5). This result may be explained due to the absence of a greenfinch competitor species on Gran Canaria. This scenario is also supported by the fact that the highest values of phenotypic variation (standard error) of bill depth and width in *F. coelebs* and *F. teydea* populations are reported on Gran Canaria [13].

We have interpreted and discussed hitherto ancient ecological interactions between extinct and extant finch species. However, all greenfinch species are now extinct and it is plausible to think that the extant finch species could have modified their bill morphology since the extinction. This question is difficult to answer without fossil record of the extant species of Macaronesian chaffinches. If the bill morphology of *F. coelebs* from Madeira and the Canary Islands changed after the greenfinch extinctions due to character release, we should expect a similar morphology to those of Azores, at least in the islands without *F. teydea* (i.e. Madeira, La Gomera, La Palma and El Hierro). In the same way, we expect a similar beak size and shape in both populations of *F. teydea*. However, that pattern is not observed. Rodents may be especially important competitors to finches for large seeds [2]. Thus, the high densities of alien rodents recorded in the Canary Islands forest [37] could explain the absence of these patterns in chaffinch populations. Future studies will be necessary to test this hypothesis.

### Conclusion

The finding of a new greenfinch species not only represents the discovery of a new and recently extinct bird, but it also provided an unexpected opportunity for studying ancient ecological interactions that have been as yet poorly understood. This study indicates that *C. aurelioi*, *F. coelebs* and *F. teydea* co-occurred in Tenerife forests in space and time. Regardless, the morphological patterns described among extant and extinct species do not definitely demonstrate character displacement, the differentiation in beak morphology among these sympatric species co-occurring in space and time provides plausible evidence that resource competition produced such divergence, resulting in ecological niche divergence. Character displacement might have been a common process in Macaronesia. Smaller bills and reduced bill size variance in the Canary and Madeira *F. coelebs* compared to the Azores also indicate divergence in beak size due to recent sympatric co-existence of several finch species exploiting similar resources. These results provide evidence that Macaronesian greenfinches experienced an unknown but significant differentiation process in the past with ecological interactions driven by competition with sympatric chaffinches. Such a system with extinct and extant species prompt a re-thinking of the evolutionary and biogeographic history of Macaronesian finches, where extinct birds are key to understanding the morphological and phenotypic variation of extant finches in the region.

## Materials and Methods

### Fossil material

Fossil material was collected in Cueva del Viento, a 17 km long complex system of volcanic lava tube galleries formed 0.17–0.13 My ago [38], [39], and situated in the north side of Tenerife (Canary Islands; Figure S1). Specifically, bones were collected in a remote gallery called “Galería de los Pájaros”. Before picking up the material, locations of bird, mammal and lizard specimens distributed throughout the gallery were plotted on a topographic map. Afterwards, they were collected from the cave floor. Many remains were found together, suggesting an absence of movement after death [32]. The former entrance to this part of the cave is 700 meters above sea level (UTM X: 333122; Y: 3137145). This entrance is now blocked by rocks and sediments. The distance of the fossil site to the current entrance of the cave (more than 1 km) makes it difficult for people to access, resulting in exceptionally good preservation of the bones. All specimens from this study, except one used for radiocarbon dating, were consolidated with paraloid B72 synthetic resin diluted in acetone (10%).

We compared the fossil material with recent skeletons curated in the Institut Mediterrani d'Estudis Avançats from the Balearic Islands (IMEDEA), and from the vertebrate collection of Departamento de Zoología in La Laguna University (DZUL) from the Canary Islands. Skeletons used for these comparisons are listed in Appendix S1. Anatomical terminology follows [40]–[42].

### Morphological analysis

We took measurements (1–23) with digital callipers to the nearest 0.01 mm, as shown in Figure S6. In order to measure the area of the *carina* (to the intersection with the main body of sternum) and the *rostrum sterni* (measure 24), we photographed the bones with a digital camera (Nikon Coolpix 5900) at the same distance and orientation. The areas have been obtained with the program analySIS®, (Soft Imaging System GmbH, http://www.soft-imaging.net). For further details on image preparation and measurements see [43].

We examined for morphological differences between *C. aurelioi* and *C. chloris* performing a multivariate analysis of variance (MANOVA) [44] using the morphological traits measured (Table 1). Three additional MANOVAs were performed: (i) using beak and cranial variables (traits 1–5, 7–12) of *C. aurelioi*, *F. teydea teydea* and *F. coelebs canariensis* to test morphological differentiation among the sympatric seed eating forest birds from Tenerife; (ii) using beak variables (traits 1–3) in order to test morphological differentiation between the two subspecies of *F. teydea*; and (iii) test morphological beak differentiation between *C. aurelioi* and *F. teydea polatzeki* (traits 1–3). Variables were log_10_ transformed in order to meet normality and homogeneity of variances. Those variables that did not meet parametric assumptions were compared by Mann-Whitney U tests. The Bonferroni's correction (0.05/test numbers) [45] was used to avoid type I errors. Finally, we reduced the number of cranial and beak traits of the three sympatric seed eating forest birds co-occurring in Tenerife (i.e. *C. aurelioi*, *F. teydea* and *F. coelebs*) using a Principal Component Analyses (PCA). Our main interest is summarized the foraging segregation among the sympatric granivores species of Tenerife based on a small number of components [46]. We performed all statistical analyses with SPSS 17.0.

In order to estimate the mass of C. aurelioi we used the expression Y = 1.05 ∗ X^0.326^ [47], where the tibiotarsus length was the dependent variable (Y), and the estimated mass of the bird (X) was the independent variable. Only complete tibiotarsi were used.

In order to evaluate the flight capability of the new species we measured the wing lengths and areas (to calculate wing loading) [48] of fresh specimens of selected Fringillidae. We prepared the skeletons of these birds by maceration, and afterwards the forelimb bones were measured. We performed two linear regressions from these specimens: (1) length of forelimb bones (humerus+ulna+capometacarpus) versus wing length, with wing length as the dependent variable, and length of forelimb bones as the independent variable; and (2) forelimb bones length versus wing area, with wing area as the dependent variable and the forelimb bones length the independent variable.

### Dating fossil material

We dated bones by accelerator mass spectrometer radiocarbon analysis (AMS ^14^C). The collagen of the following bones, from a single skeleton, was used: fragments of braincase, maxilla and mandible, humeri and femora, right coracoid and ulna. We expressed the ^14^C age as 2σ intervals (i.e., *p* = 95.45%), and its interpretation is based exclusively on the extreme values of this interval (in order to have a *p*>95.45% that the true age of the dated material is more recent than the lower extreme value of the 2σ interval, and more ancient than the upper extreme of this interval) [49]–[53]. We present dates coming from the calibration of radiometric results as ‘cal yr BP’, and calibrate the radiocarbon following the program OxCal v4.1.3 [54] using IntCal04 calibration curve.

### Phylogenetic analyses

We used blood samples and cytochrome b (cyt b) sequences available from Genbank (Table S1) from all subspecies in order to estimate phylogenetic relationships and time of colonization of *F. teydea* and *F. coelebs* in Macaronesia. Additionally, we obtained a blood sample from *C. chloris* and cyt b sequences from *F. montifringilla* (branbling) available from Genbank for use as outgroups. From blood samples a region of the cyt b gene was amplified using primers and PCR conditions used by [55]. We aligned all sequences by eye using BioEdit [56] and taking as reference common chaffinch sequences available from Genebank. Uncorrected pairwise genetic distances among taxa were conducted using MEGA 4.0 [57]. Phylogenetic relationships between island chaffinch populations were obtained by Bayesian inference (BI) using Mr. Bayes v. 3.1.2 [58], [59]. The optimal maximum likelihood model was determined using the Akaike Information Criterion implemented in the program Modeltest 3.07 [60]. Four heated Markov chains were run for 10 million generations with trees sampled every 100 generations. The first 25,000 trees were discarded (burn-in period), and the remaining trees were used to estimate posterior probabilities of tree topology. Because the default temperature for chain heating (t = 0.20) did not allow switching among chains, the temperature was lowered to t = 0.15 in order to increase the likelihood of a switch being accepted. Four independent runs were performed to ensure the posterior probabilities were stable.

Divergence times between and within Macaronesian chaffinches were estimated using the program BEAST v. 1.5.2 [61]. We used an uncorrelated lognormal relaxed model and a Yule tree prior. We allowed the substitution rate to vary between branches with a normal distribution, with a mean of 0.01 and standard deviation of 0.0075 substitutions per site per million years corresponding to an average substitution rate of 2% and sequence divergence rate between 0.5% and 3.5% per million years [62]. The best nucleotide substitution model was inferred by hierarchical likelihood ratio tests from Modeltest 3.07. The model and values obtained were then used in the BEAST analysis. We used a Markov Chain Monte Carlo (MCMC) chain length of 50,000,000 with a burning of 1,000,000 and parameters logged every 1,000 trees. We performed two independent analyses and convergence of the chains was assessed with TRACER v.1.4.1.

### Nomenclatural Acts

The electronic version of this document does not represent a published work according to the International Code of Zoological Nomenclature (ICZN), and hence the nomenclatural acts contained in the electronic version are not available under that Code from the electronic edition. Therefore, a separate edition of this document was produced by a method that assures numerous identical and durable copies, and those copies were simultaneously obtainable (from the publication date noted on the first page of this article) for the purpose of providing a public and permanent scientific record, in accordance with Article 8.1 of the Code. The separate print-only edition is available on request from PLoS by sending a request to PLoS ONE, 185 Berry Street, Suite 3100, San Francisco, CA 94107, USA along with a check for $10 (to cover printing and postage) payable to “Public Library of Science”.

In addition, this published work and the nomenclatural acts it contains have been registered in ZooBank, the proposed online registration system for the ICZN. The ZooBank LSIDs (Life Science Identifiers) can be resolved and the associated information viewed through any standard web browser by appending the LSID to the prefix http://zoobank.org/. The LSID for this publication is: urn:lsid:zoobank.org:pub:0D157B89-93D2-47CA-B8C1-D7C9F8818FDE.

## Supporting Information

Figure S1 Geographic situation of Macaronesia. Distribution of extant species and subspecies of chaffinches (genus Fringilla), and extinct (†) greenfinches (genus Carduelis) in Macaronesia.
(0.44 MB TIF)

Figure S2 Principal Component Analysis (PCA) of Tenerife finches. PCA plot for the two principal components obtained from measurements of cranial and beak traits of Fringilla teydea teydea, F. coelebs canariensis and Carduelis aurelioi from Tenerife.
(0.55 MB TIF)

Figure S3 Tree topology obtained from Bayesian Inference, pathway and time of colonization for Macaronesian chaffinches. (A) Tree topology from Bayesian inference. Numbers above nodes show Bayesian posterior probability support 0.7. Numbers below nodes indicate mean estimated time (in million of years) of the most recent common ancestor estimated from BEAST. Lower and upper 95% highest posterior density values are also presented in brackets. Coelebs: Fringilla coelebs. Teydea: F. teydea. EH: El Hierro. LP: La Palma. LG: La Gomera. TF: Tenerife. GC: Gran Canaria. MD: Madeira. IP: Iberian Peninsula. MO: Morocco. AZ: Azores. (B) Pathways of colonization based on [16] and our own data. Mean estimated time, lower and upper 95% values, for each colonization event, are given in brackets.
(0.72 MB TIF)

Figure S4 Tenerife forest finches (Canary Islands). Bill dimensions (mean ± SD) of extant and extinct (†) Tenerife forest finches (Canary Islands). (A) Fringilla teydea teydea; (B) Carduelis aurelioi; (C) F. coelebs canariensis. Scale = 2 cm.
(2.44 MB TIF)

Figure S5 Beak size variation among Macaronesian chaffinches. Beak dimensions (mean ± standard error in mm) of Fringilla coelebs and F. teydea populations from Macaronesia. Beak depth, width and length of males are showed. For Azores and Canary Islands F. coelebs populations data from the bigger and smaller populations are showed. Data from [13].
(0.22 MB TIF)

Figure S6 Measurements. Diagram showing measurements used in this paper. Bones are not to scale. 1: maxilla length; 2: maxilla width; 3: maxilla height; 4: interorbital width; 5: cranium width; 6: cranium length; 7: cranium height; 8: mandible length; 9: symphysis length; 10: mandible width; 11: articular end width; 12: mandible height; length of 13: scapula; 14: coracoid; 15: humerus; 16: ulna; 17: carpometacarpus; 18: femur; 19: tibiotarsus; 20: tarsometatarsus; 21: basal phalax of dig I; 22: sternum; 23: carina sterni height; and 24: area of carina and rostrum sterni.
(0.17 MB TIF)

Table S1 List of living taxa used in the phylogenetic analyses and Genbank accession numbers.
(0.04 MB DOC)

Table S2 Uncorrected pairwise sequence divergences (%) within and between finches taxa analysed.
(0.06 MB DOC)

Appendix S1 Comparative material examined.
(0.02 MB DOC)
