# Supplementary figures and images for: Disentangling Ancient Interactions: A New Extinct Passerine Provides Insights on Character Displacement among Extinct and Extant Island Finches
Source: PLoS One. 2010 Sep 23;5(9):e12956. doi: 10.1371/journal.pone.0012956 (PMC2944890; doi:10.1371/journal.pone.0012956)

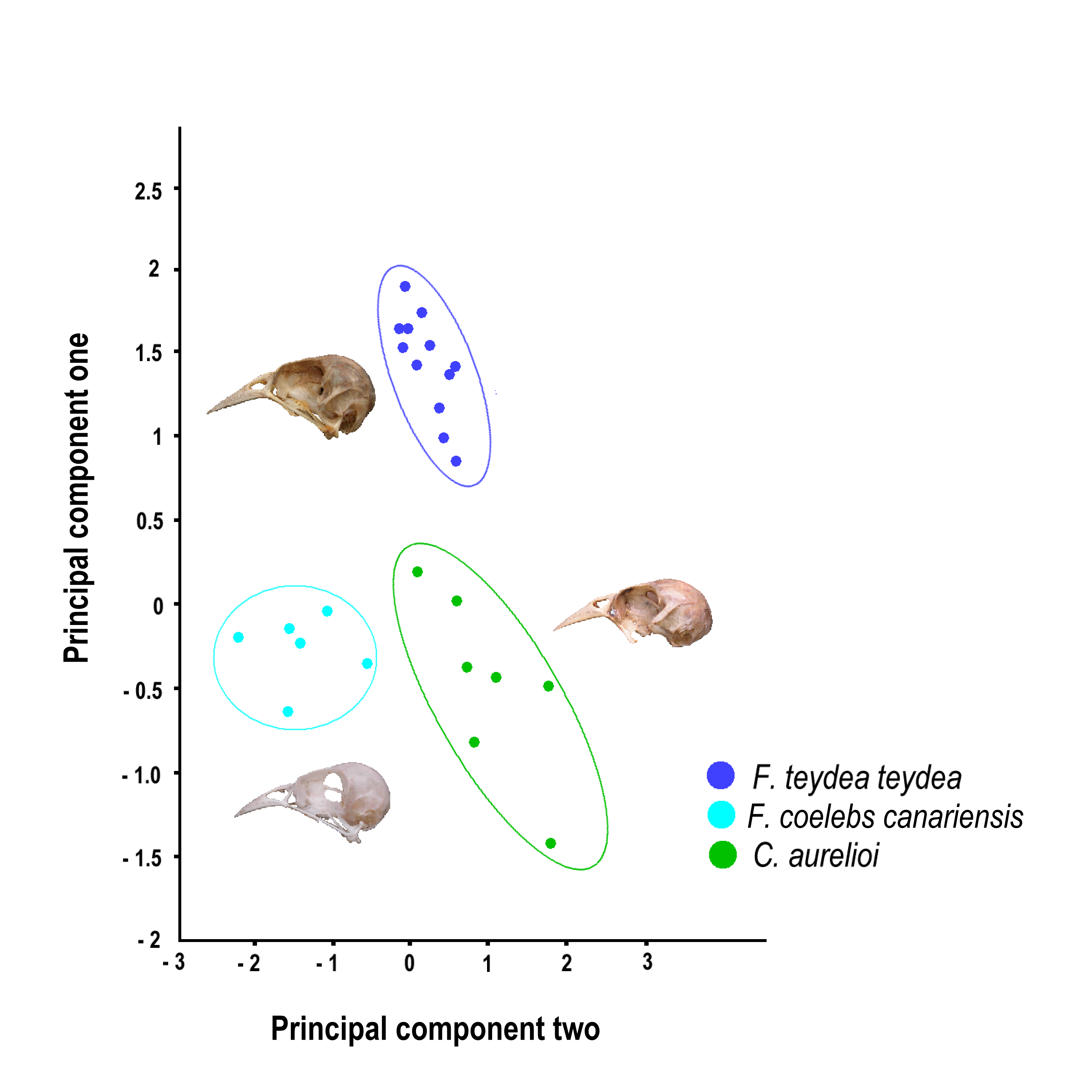

Supplement: Figure S2 — Principal Component Analysis (PCA) of Tenerife finches. PCA plot for the two principal components obtained from measurements of cranial and beak traits of Fringilla teydea teydea, F. coelebs canariensis and Carduelis aurelioi from Tenerife. (0.55 MB TIF) [file pone.0012956.s002.tif]

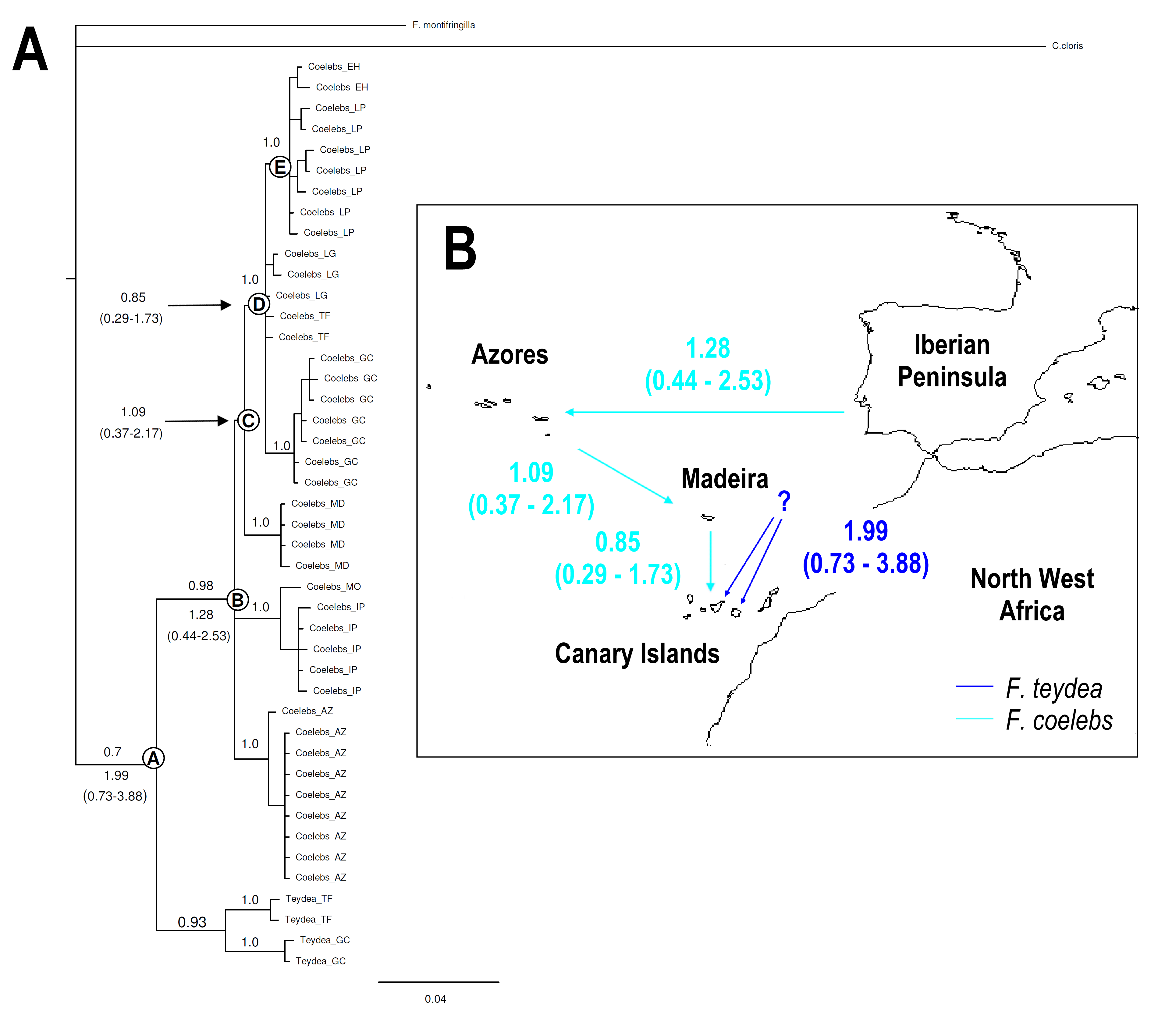

Supplement: Figure S3 — Tree topology obtained from Bayesian Inference, pathway and time of colonization for Macaronesian chaffinches. (A) Tree topology from Bayesian inference. Numbers above nodes show Bayesian posterior probability support 0.7. Numbers below nodes indicate mean estimated time (in million of years) of the most recent common ancestor estimated from BEAST. Lower and upper 95% highest posterior density values are also presented in brackets. Coelebs: Fringilla coelebs. Teydea: F. teydea. EH: El Hierro. LP: La Palma. LG: La Gomera. TF: Tenerife. GC: Gran Canaria. MD: Madeira. IP: Iberian Peninsula. MO: Morocco. AZ: Azores. (B) Pathways of colonization based on [16] and our own data. Mean estimated time, lower and upper 95% values, for each colonization event, are given in brackets. (0.72 MB TIF) [file pone.0012956.s003.tif]

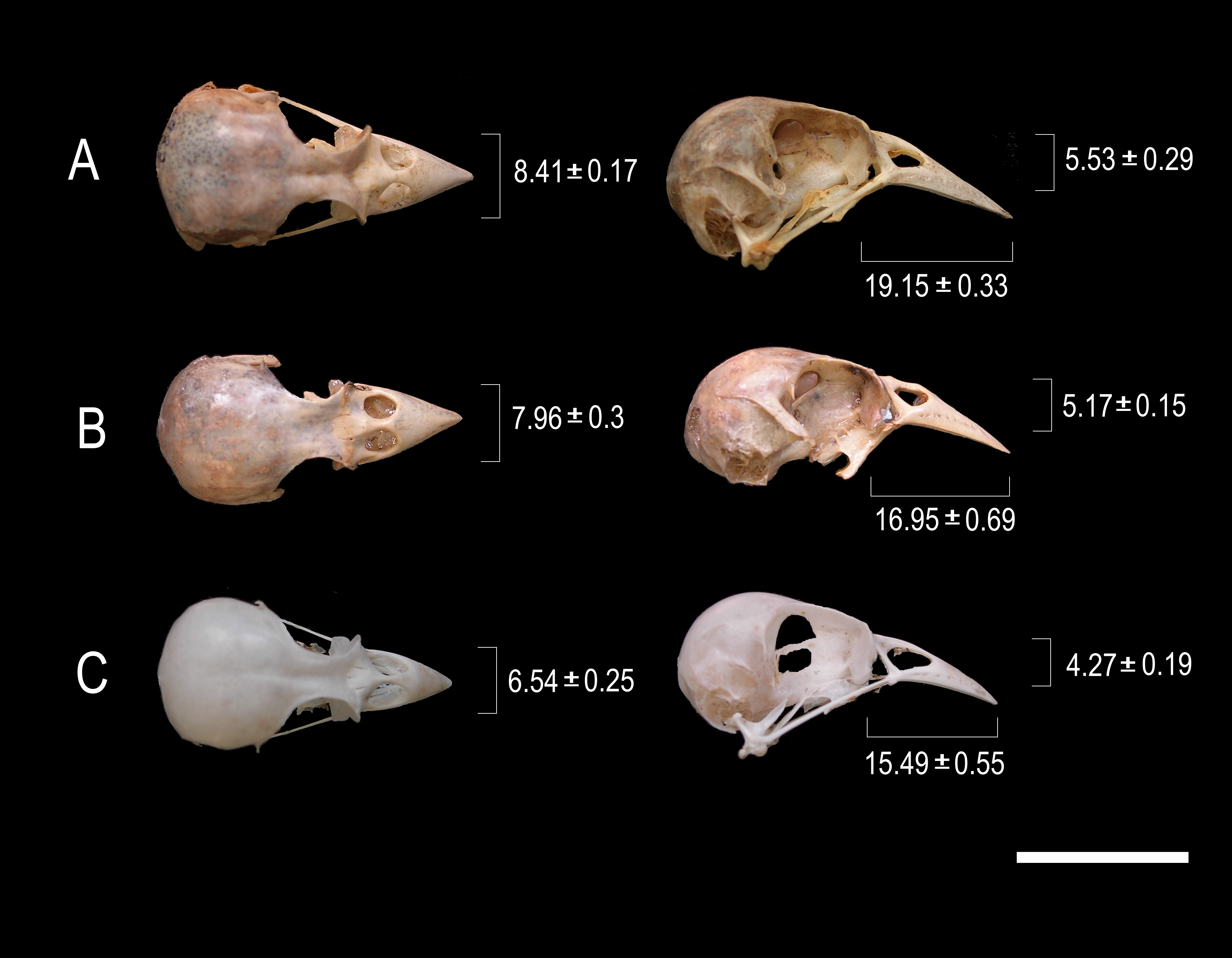

Supplement: Figure S4 — Tenerife forest finches (Canary Islands). Bill dimensions (mean ± SD) of extant and extinct (†) Tenerife forest finches (Canary Islands). (A) Fringilla teydea teydea; (B) Carduelis aurelioi; (C) F. coelebs canariensis. Scale = 2 cm. (2.44 MB TIF) [file pone.0012956.s004.tif]
