# Supplementary material for: Disentangling Ancient Interactions: A New Extinct Passerine Provides Insights on Character Displacement among Extinct and Extant Island Finches
Source: PLoS One. 2010 Sep 23;5(9):e12956. doi: 10.1371/journal.pone.0012956 (PMC2944890; doi:10.1371/journal.pone.0012956)
Supplement: Table S1 — List of living taxa used in the phylogenetic analyses and Genbank accession numbers. (0.04 MB DOC) [file pone.0012956.s007.doc]

**Table S1**. List of living taxa used in the phylogenetic analyses and Genbank accession numbers.

| Species | Subspecies | Origin | Genbank accession number |
| --- | --- | --- | --- |
| *Fringilla coelebs* | *ombriosa* | El Hierro/CI | GQ330543-44 |
|  | *palmae* | La Palma/CI | GQ330545; GQ330549-54 |
|  | *canariensis* | La Gomera/CI | GQ330546-48 |
|  | *canariensis* | Tenerife/CI | GQ330555-56 |
|  | *canariensis* | Gran Canaria/CI | GQ330557-59; GU592658* |
|  | *maderensis* | Madeira/MD | GU592659*; GU59260* |
|  | *moreletti* | Terceira/AZ | GU592661*; GU592662* |
|  | *coelebs* | IP | GU592663-66* |
|  | *africana* | Rabat/MO | GU592667* |
| *Fringilla teydea* | *teydea* | Tenerife/CI | GQ330560-61 |
|  | *polatzeki* | Gran Canaria/CI | GQ330562-63 |
| *Fringilla montifringilla* |  | unknown | AY495390 |
| *Carduelis chloris* | *aurantiventris* | Rabat/MO | GU592668* |

Islands and archipelagos included in the BI and BEAST analyses. CI: Canary Islands. MD: Madeira archipelago. AZ: Azores archipelago. IP: Iberian Peninsula. MO: Morocco. *: new haplotypes.
