# Supplementary material for: Disentangling Ancient Interactions: A New Extinct Passerine Provides Insights on Character Displacement among Extinct and Extant Island Finches
Source: PLoS One. 2010 Sep 23;5(9):e12956. doi: 10.1371/journal.pone.0012956 (PMC2944890; doi:10.1371/journal.pone.0012956)
Supplement: Table S2 — Uncorrected pairwise sequence divergences (%) within and between finches taxa analysed. (0.06 MB DOC) [file pone.0012956.s008.doc]

**Table S2.** Uncorrected pairwise sequence divergences (%) within and between finches taxa analysed.

|  | **F.c.o.** | **F.c.p.** | **F.c.c. (LG)** | **F.c.c. (TF)** | **F.c.c. (GC)** | **F.c.ma.** | **F.c.mo.** | **F.c.c.** | **F.c.a** | **F.t.t.** | **F.t.p.** | **F.m.** |
| --- | --- | --- | --- | --- | --- | --- | --- | --- | --- | --- | --- | --- |
| *F.c.ombriosa* |  |  |  |  |  |  |  |  |  |  |  |  |
| *F.c.palmae* | 0.4 |  |  |  |  |  |  |  |  |  |  |  |
| *F.c.canariensis (LG)* | 0.7 | 0.7 |  |  |  |  |  |  |  |  |  |  |
| *F.c.canariensis (TF)* | 0.7 | 0.7 | 0.1 |  |  |  |  |  |  |  |  |  |
| *F.c.canariensis (GC)* | 1.0 | 1.0 | 0.8 | 0.8 |  |  |  |  |  |  |  |  |
| *F.c.maderensis* | 1.4 | 1.4 | 1.1 | 1.0 | 1.5 |  |  |  |  |  |  |  |
| *F.c.moreletii* | 1.7 | 1.6 | 1.4 | 1.2 | 1.7 | 1.5 |  |  |  |  |  |  |
| *F.c.coelebs* | 2.0 | 1.9 | 1.7 | 1.5 | 2.0 | 1.8 | 1.8 |  |  |  |  |  |
| *F.c.africana* | 1.9 | 1.8 | 1.4 | 1.3 | 1.8 | 1.7 | 1.7 | 0.6 |  |  |  |  |
| *F.t.teydea* | 4.0 | 3.8 | 3.7 | 3.5 | 4.1 | 3.2 | 3.7 | 3.9 | 3.9 |  |  |  |
| *F.t.polatzeki* | 4.0 | 3.8 | 3.9 | 3.7 | 4.0 | 3.5 | 3.9 | 4.0 | 4.0 | 1.8 |  |  |
| *F. montifringilla* | 6.7 | 6.6 | 6.2 | 6.0 | 6.7 | 6.4 | 6.6 | 6.5 | 6.1 | 6.3 | 6.8 |  |
| *Carduelis chloris* | 10.8 | 10.6 | 10.6 | 10.4 | 10.9 | 10.7 | 10.9 | 11.0 | 11.0 | 10.6 | 10.0 | 11.9 |

F.c: *Fringilla coelebs*; F.t: *Fringilla teydea*; LG: La Gomera Island; TF: Tenerife Island; GC; Gran Canaria Island. See Figure S1 for Macaronesian chaffinches distribution. *F.c. coelebs* from Iberian Peninsula, *F. c. africana* and *C. chloris* from Morocco. Data of *F. montifringilla* from Genbank (unknown origin).
