# Supplementary material for: Disentangling Ancient Interactions: A New Extinct Passerine Provides Insights on Character Displacement among Extinct and Extant Island Finches
Source: PLoS One. 2010 Sep 23;5(9):e12956. doi: 10.1371/journal.pone.0012956 (PMC2944890; doi:10.1371/journal.pone.0012956)
Supplement: Appendix S1 — Comparative material examined. (0.02 MB DOC) [file pone.0012956.s009.doc]

Appendix SI: Comparative material examined.

At least one skeleton of all of the major genera of Passeridae and Fringillidae was examined. More thorough comparison was done with: *Carduelis triasi* † (Trias Greenfich) DZUL 1301; *C. chloris* (Greenfinch) DZUL 903, 2276, 3023 ♂, IMEDEA 2164, 9954, 12512, 12902, 12903, 12904, 12905, 12906, 12907 ♂, 12908 ♂, 12909 ♂, 12910 ♂, 12911 ♂, 12912 ♂, 12913 ♂, 12914 ♂, 12915 ♂, 12916 ♂, 12917 ♂, 20913, 48254 ♀, 48270 ♂, TFMC 35, 101 ♀; *C. cannabina* (Linnet) DZUL 573, 1537, 2294 ♂, 2864, TFMC 8, 58 ♂, 59, 72 ♀, 73 ♀, 74 ♀, 75 ♂, 76 ♀, 129 ♀, 156 ♀, 163 ♂; *C. carduelis* (Goldfinch) TFMC 82; *Serinus canarius* (Canary) DZUL 402, 584, 2985, 3024; *Fringilla coelebs* (Chaffinch) DZUL 969, 2758 ♀, 2759 ♂, 3025, 3026 ♀, 3027 ♀, 3028 ♂, 3029 ♂, 3030 ♀, 3046; *F. teydea teydea* (Blue chaffinch from Tenerife) TFMC 159 ♂, VT218 ♀, VT219 ♂, VT220 ♂; DZUL 2983, 3031 ♂, 3032 ♀, 3033 ♂, 3034 ♂, 3035 ♂, 3036 ♂, 3037 ♀, 3038 ♂, 3039 ♂; *F. teydea polatzeki* (Blue chaffinch from Gran Canaria) DZUL 3040 ♂, 3041 ♂, 3042 ♂, 3043 ♂, 3044 ♂, 3045 ♀; *Passer hispaniolensis* (Spanish Sparrow) DZUL 2278.
